# Editorial: Machine learning and data science in heart failure and stroke

**DOI:** 10.3389/fcvm.2023.1275739

**Published:** 2023-08-22

**Authors:** Leonardo Roever, Sharen Lee, Qingpeng Zhang

**Affiliations:** ^1^ Department of Clinical Research, Federal University of Uberlândia, Uberlândia, Brazil; ^2^ Gilbert and Rose-Marie Chagoury School of Medicine, Lebanese American University, Beirut, Lebanon; ^3^ Li Ka Shing Institute of Health Sciences, Faculty of Medicine, The Chinese University of Hong Kong, Hong Kong, Hong Kong SAR, China; ^4^ School of Data Science, City University of Hong Kong, Hong Kong, Hong Kong SAR, China

**Keywords:** machine learning, data science, heart failure, stroke, algorithms

**Editorial on the Research Topic** Machine learning and data science in heart failure and stroke

In this Topic on Machine Learning and Data Science in Heart Failure and Stroke, an international collection of manuscripts is presented that aims to contribute to the advancement of understanding of evidence-based approaches to the prevention, prognosis, diagnosis, and treatment of cardiovascular disease. In conclusion, the high-quality clinical case contributions presented in this Research Topic have significantly boosted knowledge, diagnosis, and treatment of cardiovascular disease in complex cases.

Liu et al. presented a retrospective study that aimed to build a machine learning (ML) model to predict the occurrence of AKI in patients with HF. The Medical Information Mart for Intensive Care-IV (MIMIC-IV) database was used. the ML model was established to predict AKI development using decision tree, random forest (RF), support vector machine (SVM), K-Nearest Neighbor (KNN) and logistic regression (LR) algorithms. Demographic, clinical, and treatment characteristics were used to establish the model. A total of 2,678 patients with HF were analyzed, 919 developed AKI. Among the 5 ML algorithms, the RF algorithm exhibited the highest performance with an AUROC of 0.96 Liu et al.

The study by Tu and collaborators aimed to identify central HF-related genes and regulatory networks using bioinformatics and validation assays. Using four sets of RNA-seq data in the Gene Expression Omnibus (GEO) database, the authors screened for HF differentially expressed genes (DEGs) using Removal of Undesired Variation from RNA-seq Data (RUVSeq) and the Robust Classification Aggregation (RRA) method.: A total of 201 robust DEGs were identified in patients with HF and NFDs. In this study identified ASPN, COL1A1 and FMOD as potential diagnostic biomarkers for HF Tu et al.

Dao and colleagues performed a systematic review of 19 studies that analyzed 5,614 participants. The objective was to compare the sensitivity and specificity of the diagnosis between the third heart sound (S3) and the left ventricular ejection fraction (LVEF) in heart failure (HF). In the result of this research, it was observed that S3 alone presented lower sensitivity in the diagnosis of HF compared to LVEF, but it was useful in the early pathological evaluation Dao et al.

Cheng et al. in their article portrayed the relationship between blood pressure patterns and age, as well as the tendency towards high prevalence of BP over time in different age groups. A total of 71,468 participants aged over 18 years with complete information on weight, height, age, gender, glucose, triglycerides, total cholesterol, systolic (SBP) and diastolic (DBP) blood pressure were included for analysis. The risk of high SBP showed a continuous increase from 35 to 79 years of age and a concomitant early increase in the risk of high DBP; after the age of 50–65 years, the high risk of PAD decreased. High-risk SBP progresses more rapidly in early life in Chinese women compared to later life Cheng et al.

Sung and colleagues developed an electronic health record based machine learning model to assess the risk of newly detected atrial fibrillation (NDAF) at an early stage after stroke. The study population consisted of a training set of 4,064 and a temporal test set of 1,492 patients. At the median follow-up of 10.2 months, the incidence rate of NDAF was 87.0 per 1,000 person-years in the test set. On the test set, the model based on both structured and unstructured data achieved a C-score of 0.840, which was significantly higher than the AS5F and CHASE-LESS scores. More studies are needed to assess the clinical utility of the prediction model Sung et al.

Huang et al. presented a retrospective study including 4,570 Chinese adults with the aim of identifying independent risk factors for carotid atherosclerosis (CAS) and constructing and validating a CAS risk prediction model based on the Chinese population. Participants were randomly assigned to the training and validation sets in a 7:3 ratio. C-index curves and receiver operating characteristics, calibration plots, and decision curve analysis (DCA) were used to assess discrimination, calibration, and clinical applicability of the risk model. In the training, internal validation, and external validation sets, the risk model showed good discriminatory power with C-indices of 0.961 (0.953–0.969), 0.953 (0.939–0.967) and 0.930 (0.920–0.94 0), respectively, and excellent calibration. The development of risk models can contribute to the early identification and prevention of CAS Huang et al.

Burton and colleagues studied 396 patients using electromechanical (EM) waveforms to assess left ventricular end-diastolic pressure elevation (LVEDP). This analysis identified subgroups of patients with varying degrees of LVEDP elevation based on waveform characteristics Burton et al.

Susic et al. used data recorded on 37 patients using two types of electronic stethoscopes. This study demonstrated that, in patients with chronic heart failure, machine learning algorithms can outperform cardiologists in detecting episodes of decompensation based on heart sounds alone Susic et al.

In the research by Sabovčik and collaborators, data from 30,354 individuals from 6 cohorts were used. The predictive performance of increased survival gradient (GBS), CoxNet, the PCP-HF risk score, and a stacking method were evaluated. In the accuracy recall (PR) analysis for predicting 10-year HF risk, the stacking method, combining the SGB, CoxNet, Gaussian mixture, and PCP-HF models, outperformed other models with PR/AUC 0.804, while PCP-HF achieved only 0.551. Flexible ML algorithms can be used to capture these diverse distributions and produce more accurate prediction models Sabovčik et al.

Gtif and colleagues used data from 116 patients with heart failure with the pathogenesis of reduced ejection fraction (HFrEF). A generalized linear model (GLM), random forest, and extreme gradient augmentation models were developed to predict the risk of post-discharge mortality using clinical and laboratory data. The result obtained was a discriminatory power of 74.5% for post-mortality by the area under the curve (AUC) Gtif et al.

In conclusion, this research topic presented several machine learning models that can be used to improve diagnosis and treatment in patients with stroke and heart failure. More studies are needed to improve and validate these techniques nets patient type.

